# Association between the C-reactive protein–triglyceride glucose index and coronary collateral circulation in patients with chronic total occlusion: a retrospective study

**DOI:** 10.7717/peerj.21576

**Published:** 2026-07-20

**Authors:** XueYu Lv, Zhihang Du, Xiaoyan Lu, Xu Liu, Dan Li

**Affiliations:** 1 Department of Cardiology, The Affiliated Hospital of Qingdao University, Qingdao, China; 2 Department of Nephrology, The Affiliated Hospital of Qingdao University, Qingdao, China; 3 Department of Radiology, The Affiliated Hospital of Qingdao University, Qingdao, China

**Keywords:** Chronic total occlusion, Coronary collateral circulation, CTI index, Risk prediction, Biomarker, Insulin resistance, Systemic inflammation

## Abstract

**Objective:**

To examine the association between the C-reactive protein-triglyceride glucose (CTI) index and coronary collateral circulation (CCC) in patients with chronic total occlusion (CTO), and to assess its predictive performance for poor CCC compared to traditional biomarkers, by developing an accessible risk stratification tool based on routine clinical data, we seek to facilitate early identification of high-risk patients and inform targeted clinical management.

**Methods:**

This retrospective study analyzed 545 patients, classifying CCC using the Rentrop score. The CTI index was calculated from high-sensitivity C-reactive protein (hs-CRP), triglycerides, and fasting plasma glucose. The association between CTI (as a continuous variable) and poor CCC was assessed using multivariate logistic regression, with results presented as odds ratios (OR) and 95% confidence intervals (CI). Model performance was evaluated by the area under the receiver operating characteristic curve (AUC). Restricted cubic spline and subgroup analyses were performed to examine the dose-response relationship and the consistency of the association, respectively.

**Results:**

Among the 545 CTO patients, 167 (30.6%) developed poor CCC. The poor CCC group demonstrated significantly higher levels of CTI, inflammatory markers, and cardiometabolic risk indicators (all *P* < 0.001). Multivariable logistic regression revealed the CTI index as an independent risk factor for poor CCC (OR = 2.63, 95% CI [1.75–4.10], *P* < 0.001). The receiver operating characteristic (ROC) analysis showed the CTI index (AUC = 0.679, 95% CI [0.632–0.726]; *P* < 0.001) had comparable predictive ability to the triglyceride-glucose (TyG) index, atherogenic index of plasma (AIP), and neutrophil to high-density lipoprotein ratio (NHR). Restricted cubic spline analysis indicated a significant linear dose-response relationship between CTI and poor CCC risk (P-overall <0.001). Subgroup analyses confirmed the consistent association of CTI with poor CCC across various patient populations (P for interaction >0.05).

**Conclusion:**

The CTI index demonstrated a significant and independent association with impaired coronary collateral circulation in CTO patients, exhibiting modest predictive value (AUC = 0.679) compared to traditional biomarkers. This readily accessible index may serve as a complementary tool for early risk stratification. Future multi-center prospective studies are warranted to validate its clinical utility and explore underlying mechanisms.

## Introduction

In clinical cardiology, chronic total occlusion (CTO) is not uncommon in coronary artery disease. It is characterized by the complete cessation of luminal continuity within a coronary artery, persisting for a duration of at least three months (Stone et al., 2005). According to research findings, roughly 16% of patients diagnosed with significant coronary artery disease (CAD) have concurrent CTO (Råmunddal et al., 2014). When it comes to individuals undergoing invasive coronary angiography (ICA), the detection rate of CTO can reach as high as 25% (Fefer et al., 2012). Notably, this prevalence of CTO rises significantly in people who have a history of coronary artery bypass graft surgery (CABG), even approaching nearly 50% (Tomasello et al., 2015). Percutaneous coronary intervention (PCI) serves as a standard therapy for CTO, and it enhances angina relief and quality of life for CTO patients based on a randomized Euro trial (Mashayekhi et al., 2017). A meta-analysis showed that CTO-PCI improved long-term survival in patients compared with optimal medical therapy (Holck et al., 2024). But during PCI, those patients who suffer CTO have a lower success rate, a longer and more costly procedure time, a higher risk of procedural complications, which all lead to unfavorable effects after PCI (Tomasello et al., 2015; Othman et al., 2020), and are particularly common in the elderly and those who suffer kidney disease.

Importantly, well-developed coronary collateral circulation (CCC) can substantially mitigate the adverse consequences of CTO by providing alternative blood flow to the ischemic myocardium, thereby limiting infarct size, reducing post-infarction complications, alleviating angina frequency, and lowering cardiovascular mortality. CCC comprises interlinking arteriolar channels connecting epicardial coronary arteries (Seiler et al., 2013). Well-developed collaterals limit myocardial infarct size post-acute coronary occlusion, mitigate post-infarction complications, alleviate angina frequency, and lower cardiovascular plus total mortality rates (Charney & Cohen, 1993). Prior studies have found an implicit correlation between the growth of CCC and both insulin resistance (IR) and the systemic inflammatory response (Rakhit et al., 2005; Fefer et al., 2012). Furthermore, IR and its associated metabolic abnormalities can lead to endothelial dysfunction and elevate the levels of angiogenesis inhibitors, thereby impairing the formation of collateral circulation (Cao et al., 2025). Therefore, there is a need to find a biomarker that integrates inflammation and IR to assess or predict CCC formation. The high insulin-euglycemic clamp (HEC) is the gold standard for assessing IR, but it costs more and needs multiple venepunctures. Based on former research, the TyG index, which is calculated using only triglycerides and fasting glucose (TyG index = ln[(triglycerides × fasting glucose)/2]), is considered a reliable surrogate for IR. Moreover, the TyG index has been shown to be associated with a higher risk of poor CCC in those with CTO (Gao et al., 2021; Zhu et al., 2024). C-reactive protein (CRP) has been proven to have a strong association with CHD as a classical nonspecific inflammatory biomarker (Rolver et al., 2024). Indeed, emerging evidence highlights an immuno-metabolic interplay in CCC formation. The vicious cycle between inflammation and IR establishes a feedforward loop that suppresses vascular compensation, significantly impairing coronary collateral growth (He et al., 2022; Chen et al., 2024). As a combined biological indicator, the C-reactive protein–triglyceride glucose (CTI) index is created by merging the TyG index with CRP, and its main purpose is to simultaneously reflect the body’s metabolic conditions and inflammatory status in a single measure (Ruan et al., 2022). The CTI index has shown value in assessing disease progression across multiple conditions, such as depression, cancer-related mortality and coronary artery disease, but there is no evidence yet to indicate the evaluative value of CTI for the CCC. Therefore, this research intends to examine the potential association between CTI values and CCC status in CTO-affected individuals as an initial investigation.

## Materials & Methods

### Study population

**Figure 1:**
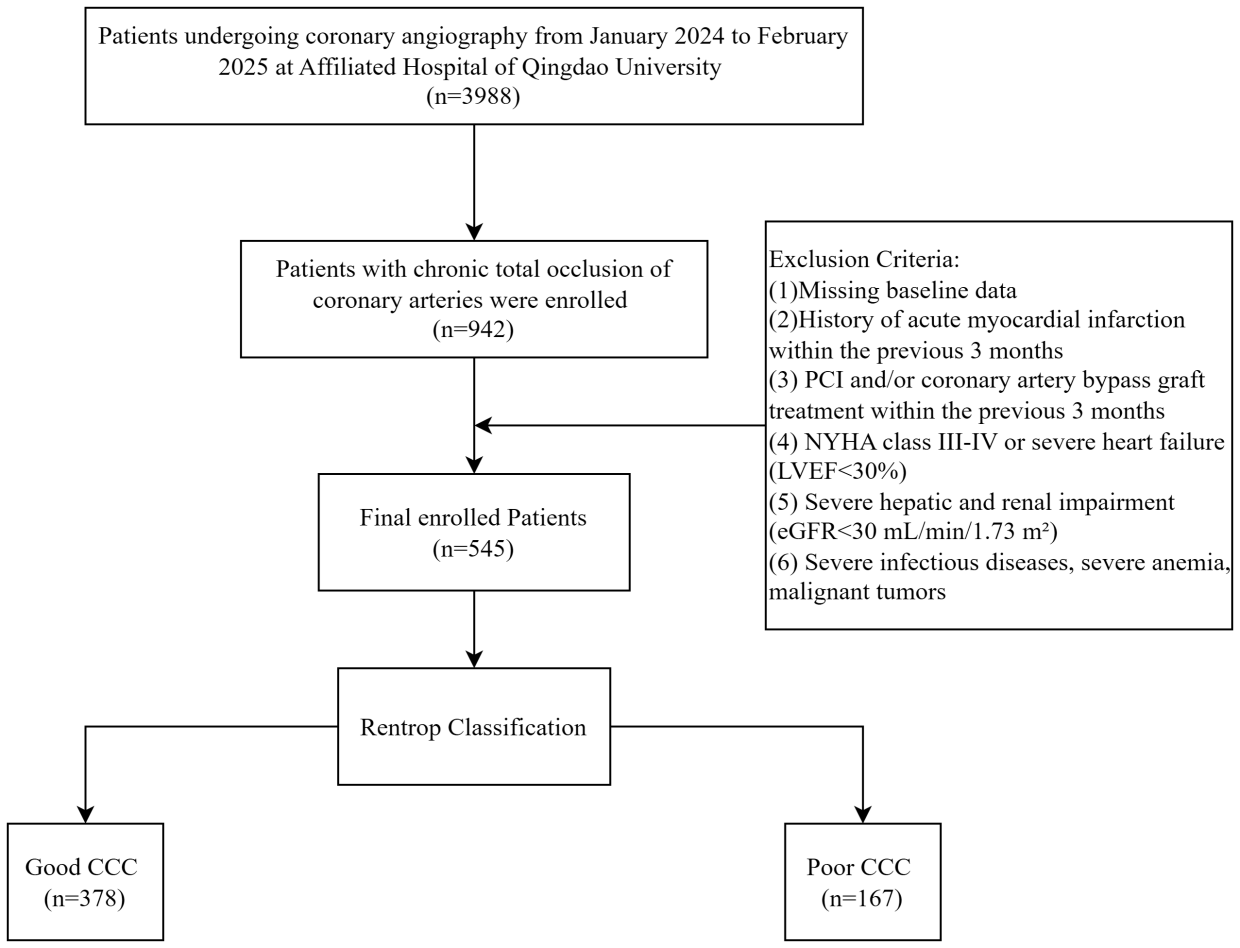
Flow chart of patient recruitment.

We enrolled 3,988 patients in this study, and a total of 545 patients with CTO in one or more major coronary branches were included as illustrated in Fig. 1. Given the single-center, observational, and cross-sectional characteristics of this study, all patients included were confirmed *via* coronary angiography at the Affiliated Hospital of Qingdao University from January 2024 to February 2025. The specific exclusion criteria for all included patients were as follows: (1) NYHA classification III-IV (to exclude patients with severe heart failure that could independently impair collateral development); (2) incomplete baseline clinical data (to ensure completeness and accuracy for CTI calculation and multivariable adjustment); (3) previous receipt of PCI and/or coronary artery bypass grafting (CABG) within 3 months (to avoid confounding effects from recent revascularization procedures); (4) acute myocardial infarction (AMI) occurring in the past 3 months (to exclude acute inflammatory states that could transiently affect CTI values and CCC assessment); (5) severe hepatic or renal dysfunction or left ventricular ejection fraction (LVEF) <30% (to avoid severe comorbidities known to markedly influence inflammatory and metabolic markers); (6) active severe infections, severe anemia, or malignant tumors (to eliminate acute or chronic conditions that could substantially elevate CRP or alter metabolic parameters). This research obtained approval from the Ethics Committee of the Affiliated Hospital of Qingdao University (Approval Number: QYFY WZLL 30723) and adhered strictly to the Declaration of Helsinki. Given that this study adopted a cross-sectional research design, the Ethics Committee referenced above has waived the requirement to obtain informed consent from participants. All personal identifying information of the patients was fully anonymized to protect privacy.

### Data collection and delineation

The key baseline data, including name, gender, age, medical history, alcohol consumption history, and other relevant details, were collected for all patients fulfilling the inclusion criteria. Peripheral venous blood samples were obtained by clinical staff in the morning after hospital admission, after an overnight fasting period of at least 8 h. The blood specimens were assayed using internationally standardized methods and instruments to determine their concentrations. The CTI index was calculated by the standard formula: CTI index = 0.412  × Ln (hs-CRP [mg/L]) + Ln [(TG [mg/dL] × FPG [mg/dL])/2]. The AIP index was calculated as Ln (TG [mg/dL]/HDL-C [mg/dL]). The NHR index was calculated as neutrophil count (×10^9^/L) divided by HDL-C (mg/dL). To further clarify the anatomical characteristics of coronary arteries, all participants underwent coronary angiography (CAG) *via* the radial or femoral arterial approach, based on their individual clinical conditions, using the standard Judkins method. CCC was categorized using the Rentrop scoring criteria, with specific grades defined as follows: Rentrop grade 0: Absence of detectable collateral circulation perfusion; Rentrop grade 1: Collateral flow reaching the branches of the target vessel, without visualization of the epicardial segment; Rentrop grade 2: Partial collateral perfusion of the epicardial segment of the target vessel; Rentrop grade 3: Complete collateral perfusion of the target vessel’s epicardial segment. All angiograms were independently evaluated by two experienced interventional cardiologists who were blinded to the patients’ laboratory findings, including CTI values and other biochemical markers. In cases of disagreement, a third senior cardiologist reviewed the angiogram and a consensus grade was reached. The highest Rentrop grade among all involved side branches was adopted if patients suffered multiple coronary artery lesions. Based on the Rentrop grading results, we stratified patients into two groups: the poor CCC group (Rentrop grades 0–1) and the good CCC group (Rentrop grades 2–3). Additionally, we stratified patients into subgroups based on indicators such as age, gender, and estimated glomerular filtration rate (eGFR) for subsequent subgroup analysis.

### Statistical analysis

**Figure 2:**
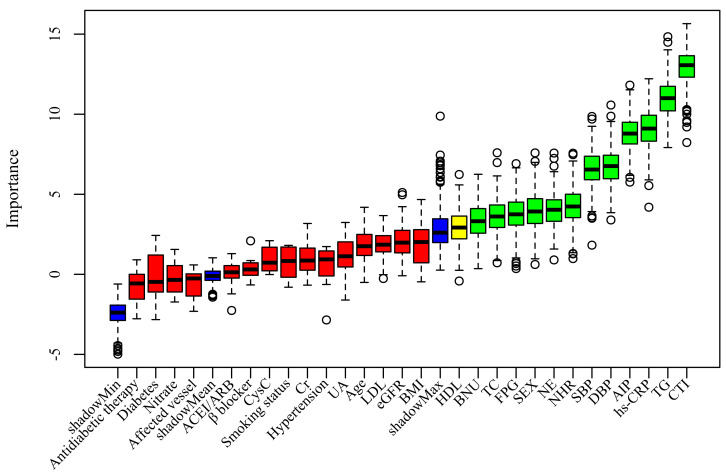
Feature selection process for CCC based on Boruta’s algorithm. Boxplots display the Random Forest importance scores (Z-scores) for candidate features. Green, red, and yellow denote confirmed, rejected, and tentative features, respectively. Blue boxplots represent randomized shadow features used as significance thresholds. BMI, body mass index; FPG, fasting plasma glucose; TC, total cholesterol; TG, triglycerides; SBP, systolic blood pressure; DBP, diastolic blood pressure; hs-CRP, high-sensitivity C-reactive protein; ACEI/ARB, angiotensin-converting enzyme inhibitors/angiotensin receptor blockers.

This study used the Kolmogorov–Smirnov test to evaluate the normality of data and the Levene test to assess the homogeneity of variance for normally distributed continuous variables. The independent samples *t*-test was applied for intergroup comparisons if variance homogeneity was confirmed; otherwise, Welch’s *t*-test was utilized. Normally distributed continuous variables were expressed as the mean ± standard deviation (SD), and continuous variables failing to follow a normal distribution were documented as medians plus interquartile ranges (IQR), and the Mann–Whitney U test was used to compare data across different groups. For categorical variables, the corresponding data were shown as frequencies (with percentages in parentheses), and the Pearson’s chi-squared test was employed to compare differences between groups. We employed a parallel modeling approach to investigate the independent associations of the CTI index, as well as the TyG, AIP, and NHR indices, with poor CCC. For each biomarker, three separate logistic regression models were established: Model 1 was unadjusted. Model 2 was adjusted for baseline demographic and clinical factors: age, sex, body mass index (BMI), hypertension, diabetes mellitus, and smoking status. Model 3 was built upon Model 2 by integrating additional covariates identified *via* Boruta feature selection (500 iterations). Among the 12 highly predictive features initially confirmed (Fig. 2), seven were excluded from the covariate pool to prevent multicollinearity and conceptual overlap: three alternative composite indices (CTI, AIP, NHR), three fundamental components of the CTI index (hs-CRP, TG, FPG; omitted despite VIF < 5), and sex (already in Model 2). Consequently, the remaining five variables—Blood Urea Nitrogen (BUN), Neutrophils (NE), Total Cholesterol (TC), Diastolic Blood Pressure (DBP), and Systolic Blood Pressure (SBP)—were incorporated into Model 3. This identical adjustment strategy was applied consistently across all four parallel biomarker models. Restricted cubic spline (RCS) analysis was conducted to characterize the dose–response relationship. The receiver operating characteristic (ROC) curve and area under the curve (AUC) were used to assess the sensitivity and specificity of the CTI index for predicting collateral circulation formation. To further clarify its predictive performance, the AUC of the CTI index was compared with those of the TyG, AIP, and NHR indices using pairwise comparisons. Subgroup analyses were performed to confirm the consistency of the association between the CTI index and poor CCC across various patient populations (age <60 or ≥60 years, eGFR <60 or ≥60 mL/min/1.73 m^2^, hypertension status, diabetes status, smoking status, BMI <25 or ≥25 kg/m^2^ and number of diseased vessels). All statistical analyses were implemented using R software (version 4.3.0). All tests were two-tailed, with a significance level set at *P* < 0.05; results yielding *P*-values below this threshold were considered statistically significant.

## Results

### Participant characteristics

Baseline characteristics were stratified according to collateral circulation development, yielding 167 participants in the poor CCC group and 378 participants in the good CCC group. The poor CCC group exhibited higher levels of cardiometabolic risk indicators, including the CTI index (*P* < 0.001), triglycerides (*P* < 0.001), total cholesterol (*P* = 0.009), LDL-C (*P* = 0.032), and fasting blood glucose (*P* < 0.001). Additionally, inflammatory markers—hs-CRP and neutrophil count—were markedly elevated in the poor CCC group (*P* < 0.001). Comprehensive details regarding the participants’ baseline characteristics are presented in Table 1.

**Table 1:** Basic characteristics of the population.

Characteristics	Total (*n* = 545)	Good CCC (*n* = 378)	Poor CCC (*n* = 167)	*p* value
**Demographics**				
Age, years	64.0 (54.0, 71.0)	64.5 (55.0, 72.0)	63.0 (53.0, 69.0)	0.148
Gender				<0.001
Female	127 (23.3%)	71 (18.8%)	56 (33.5%)	
Male	418 (76.7%)	307 (81.2%)	111 (66.5%)	
BMI, kg/m^2^	26.0 (24.2, 28.1)	26.0 (23.9, 28.1)	26.1 (24.3, 27.9)	0.408
SBP (mmHg)	132.0 ± 17.7	133.8 ± 17.3	128.1 ± 18.2	<0.001
DBP (mmHg)	78.0 (70.0, 85.0)	78.0 (71.0, 85.0)	76.0 (69.0, 85.0)	0.204
Current smoking	178 (32.7%)	120 (31.7%)	58 (34.7%)	0.558
**Co-morbidities (n, %)**				
Diabetes	213 (39.1%)	142 (37.6%)	71 (42.5%)	0.319
Hypertension	363 (66.6%)	250 (66.1%)	113 (67.7%)	0.803
**Medical history (n, %)**				
Antidiabetic drugs	194 (35.6%)	131 (34.7%)	63 (37.7%)	0.553
β-receptor blocker	309 (56.7%)	205 (54.2%)	104 (62.3%)	0.098
ACEI/ARB	310 (56.9%)	210 (55.6%)	100 (59.9%)	0.398
Nitrates	240 (44.0%)	172 (45.5%)	68 (40.7%)	0.345
**Laboratory measurements**				
CTI	4.8 (4.2, 5.5)	4.6 (4.1, 5.2)	5.3 (4.6, 5.7)	<0.001
TG, mmol/L	1.2 (0.9, 1.8)	1.1 (0.9, 1.6)	1.4 (1.1, 2.3)	<0.001
TC, mmol/L	4.3 (3.5, 5.2)	4.2 (3.5, 5.1)	4.6 (3.8, 5.4)	0.009
LDL-C, mmol/L	2.4 (1.8, 3.1)	2.4 (1.8, 3.0)	2.6 (2.0, 3.2)	0.032
HDL-C, mmol/L	1.3 (1.1, 1.5)	1.3 (1.1, 1.5)	1.3 (1.2, 1.5)	0.260
hs-CRP, mg/L	1.0 (0.3, 5.0)	0.6 (0.3, 4.1)	2.1 (0.5, 6.8)	<0.001
Creatinine, μmol/L	97.0 (87.0, 106.0)	98.0 (88.0, 106.0)	96.0 (85.0, 106.0)	0.493
FBG, mmol/L	5.6 (4.9, 6.7)	5.5 (4.9, 6.5)	6.0 (5.2, 7.3)	<0.001
Uric acid, mmol/L	340.0 (284.0, 405.0)	339.0 (284.0, 396.8)	343.0 (286.5, 423.0)	0.370
Urea nitrogen, mmol/L	5.9 (4.8, 7.3)	5.9 (4.9, 7.3)	5.9 (4.7, 7.5)	0.730
Neutrophils, ×10^9^/L	4.0 (3.2, 5.3)	3.9 (3.1, 5.0)	4.5 (3.5, 5.8)	<0.001
Cystatin C, mg/L	1.0 (0.9, 1.1)	1.0 (0.9, 1.1)	1.0 (0.9, 1.1)	0.965
eGFR, mL/min/1.73m^2^	67.1 (59.0, 76.0)	67.4 (59.7, 75.1)	66.5 (56.8, 78.8)	0.605
**Angiographic results**				
**Number of lesions**				0.595
One-vessel disease	238 (43.7%)	163 (43.1%)	75 (44.9%)	
Two-vessel disease	228 (41.8%)	163 (43.1%)	65 (38.9%)	
Three-vessel disease	79 (14.5%)	52 (13.8%)	27 (16.2%)	

**Notes.**

BMI body mass index SBP systolic blood pressure DBP diastolic blood pressure CTI Creactive protein–triglyceride glucose index TG triglycerides TC total cholesterol LDL-C lowdensity lipoprotein cholesterol HDL-C high-density lipoprotein cholesterol hs-CRP highsensitivity C-reactive protein FBG fasting blood glucose eGFR estimated glomerular filtration rate

### Logistic regression models for the formation of collateral circulation

Boruta feature selection (500 iterations) identified 12 highly predictive features, with the CTI index ranking top (Fig. 2). To prevent multicollinearity and conceptual overlap, alternative composite indices, their raw metabolic components, and sex were excluded. The remaining five clinical variables (BUN, NE, TC, DBP, SBP) were added to Model 2 covariates (age, sex, body mass index (BMI), hypertension, diabetes mellitus, and smoking status) to form the fully adjusted Model 3. This identical adjustment strategy was applied using a parallel modeling framework for each biomarker (Model 1: unadjusted; Model 2: baseline demographic and clinical covariates; Model 3: fully adjusted).

Parallel multivariable logistic regression (Table 2) demonstrated that the CTI index was independently associated with an increased risk of poor CCC, yielding ORs of 2.23 (95% CI [1.74–2.87]) in Model 1, 2.15 (95% CI [1.67–2.79]) in Model 2, and 2.02 (95% CI [1.55–2.64]) in Model 3 (all *P* < 0.001). In the fully adjusted Model 3, TyG (OR = 1.85, *P* < 0.001) and AIP (OR = 1.51, *P* < 0.001) also maintained significant independent associations, whereas NHR lost statistical significance (OR = 1.02, *P* = 0.157). Detailed multivariable outputs for all covariates are provided in Table S1.

**Table 2:** Associations between various biomarker indices and poor coronary collateral circulation.

	**Model 1**		**Model 2**		**Model 3**	
**Biomarker**	**OR (95% CI)**	***p* value**	**OR (95% CI)**	***p* value**	**OR (95% CI)**	***p* value**
CTI index	2.23 (1.74–2.87)	<0.001	2.15 (1.67–2.79)	<0.001	2.02 (1.55–2.64)	<0.001
TyG index	2.30 (1.75–3.06)	<0.001	2.18 (1.63–2.96)	<0.001	2.10 (1.54–2.86)	<0.001
AIP index	5.57 (2.88–11.09)	<0.001	5.68 (2.74–12.12)	<0.001	4.57 (2.13–10.07)	<0.001
NHR index	1.20 (1.08–1.34)	0.001	1.25 (1.12–1.42)	<0.001	1.22 (0.93–1.61)	0.157

**Notes.**

OR odds ratio CI confidence interval CTI C-reactive protein–triglyceride glucose index TyG triglycerideglucose AIP atherogenic index of plasma NHR neutrophil to HDL ratio

Model 1 was unadjusted.

Model 2 was adjusted for age, sex, BMI, smoking status, diabetes mellitus, and hypertension.

Model 3 was adjusted for variables in Model 2 plus BNU, NE, TC, DBP, and SBP.

### The relations between CTI and CCC

**Figure 3:**
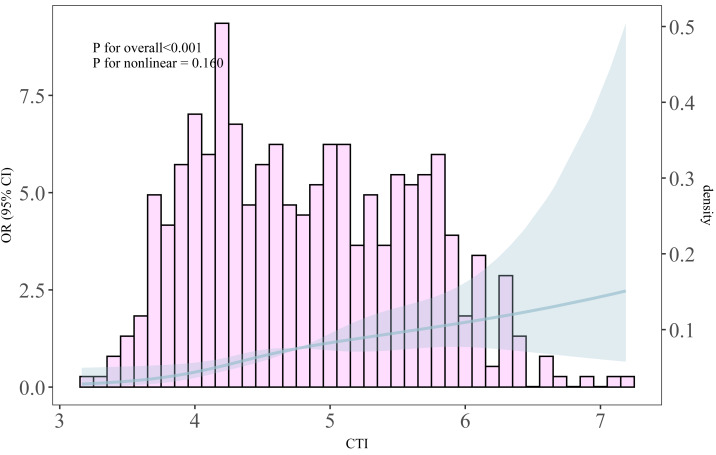
RCS analysis of CTI and the risk of poor CCC. The blue curve illustrates the nonlinear relationship between CTI and the adjusted odds ratio (OR) for poor collateral circulation, with the shaded area representing the 95% confidence interval. The underlying pink histogram shows the distribution of CTI values in the study population.

RCS analysis (Fig. 3) visually demonstrated a linear dose–response relationship between the CTI index and the risk of poor CCC, with a steady upward trend and no evidence of non-linearity (P-overall < 0.001, P-non-linearity = 0.160). These findings lend support to employing linear models for depicting the dose–response relationships between CTI index and CCC.

### CTI’s predictive performance for CCC

**Figure 4:**
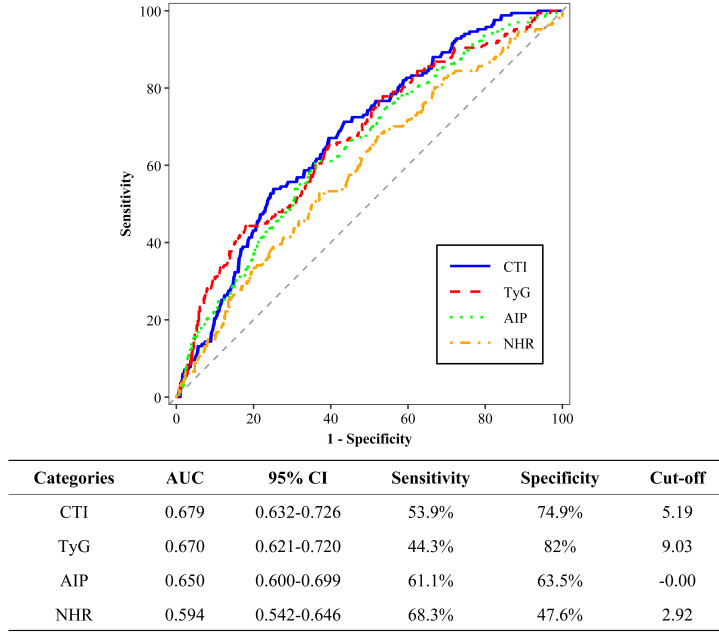
Receiver operating characteristic (ROC) curves of various biomarker indices for predicting poor coronary collateral circulation. The upper panel displays the ROC curves comparing the diagnostic performance of CTI, TyG, AIP, and NHR. The lower panel presents the detailed corresponding statistics, including the area under the curve (AUC), optimal cut-off values determined by the Youden index, and their respective sensitivity and specificity. CTI, C-reactive protein–triglyceride glucose index; TyG, triglyceride-glucose; AIP, atherogenic index of plasma; NHR, neutrophil to HDL ratio; ROC, receiver operating characteristic; AUC, area under the curve; CI, confidence interval.

ROC curve analysis demonstrated that the optimal cut-off value of the CTI index for detecting poor CCC was 5.19 (Fig. 4), with an AUC of 0.679 (95% CI [0.632–0.726]; *P* < 0.001). By comparing the results with those of the TyG, AIP, and NHR indices, it can be observed that the CTI index has comparable predictive ability. The DeLong test (Table S2) confirmed that the discriminatory ability of the CTI index was not significantly superior to that of the TyG (Difference of AUC = 0.008, *P* = 0.752) and AIP (Difference of AUC = 0.029, *P* = 0.298) indices, although it showed a statistically significant improvement over the NHR index (Difference of AUC = 0.085, *P* = 0.012).

### Subgroup analysis

**Figure 5:**
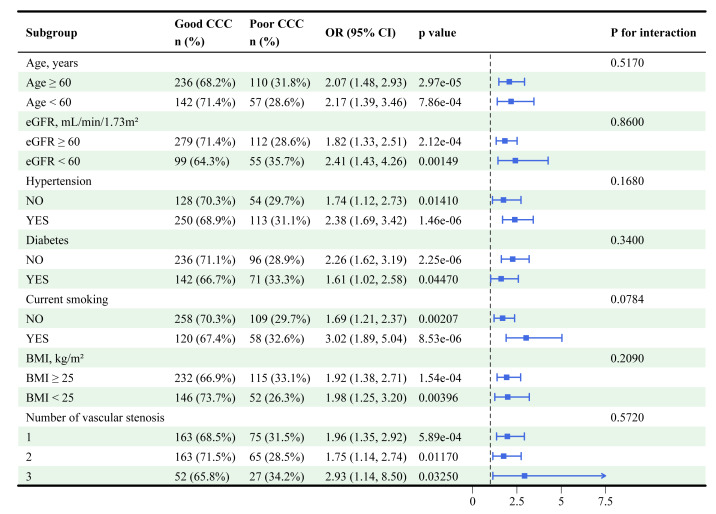
Subgroup analysis of the association between CTI and the risk of poor coronary collateral circulation. The forest plot illustrates the odds ratios (ORs) and 95% confidence intervals (CIs) for the risk of poor CCC associated with CTI across various pre-specified clinical subgroups. The P for interaction evaluates whether the predictive value of CTI differs significantly between the strata of each subgroup. CTI, C-reactive protein–triglyceride glucose index; CCC, coronary collateral circulation; OR, odds ratio; CI, confidence interval; eGFR, estimated glomerular filtration rate; BMI, body mass index.

We set different subgroups according to age (<65 or ≥ 65 years), eGFR (<60 or ≥60) diabetes status (Yes or NO), hypertension status (Yes or NO), smoking (Yes or NO), number of vascular stenosis (1 or 2 or 3), and BMI (<25 or ≥ 25 kg/m^2^). Subgroup analyses demonstrated no significant interaction effects detected across all subgroups (P for interaction > 0.05). Relevant numerical findings are presented in Fig. 5.

## Discussion

This study aimed to explore the association between the CTI index and the risk of poor CCC development in patients with CTO. Our key findings are summarized as follows: (1) In comparison to patients with persistently low CTI levels, those with elevated CTI demonstrated an increased risk of poor CCC. (2) The CTI index exhibits comparable predictive performance to other established CCC-related indicators (AUC = 0.679). Notably, a distinct linear trend was observed in the relationship between CTI and poor CCC. These findings offer robust evidence to support the potential clinical utility of the CTI index. Prior research has established that insulin resistance (IR) and chronic systemic inflammation are two interlinked pathological mechanisms that cooperatively impair coronary collateral growth. IR promotes endothelial dysfunction by inducing oxidative stress (He et al., 2006) and disrupting mitochondrial function (Muniyappa et al., 2007), while also reducing nitric oxide bioavailability (Yu, Gao & Ma, 2011) and attenuating vascular endothelial growth factor (VEGF) expression (Zou et al., 2023); concurrently, elevated C-reactive protein (CRP) exacerbates vascular injury by enhancing oxidative stress and promoting monocyte adhesion to the endothelium (Chang et al., 2002). Importantly, these two pathways operate through a bidirectional feedforward loop: pro-inflammatory cytokines impair insulin signaling, and IR, in turn, activates pro-inflammatory transcriptional programs *via* NF-κB and JNK pathways (Jia & Sowers, 2021; Li et al., 2022). This synergistic interplay provides the biological rationale for combining CRP and the TyG index into a single composite biomarker—the CTI index—to capture the combined inflammatory-metabolic burden that may compromise coronary collateral circulation. Prior studies have demonstrated that the CTI index is significantly associated with new-onset coronary heart disease (Chen et al., 2025), cardiovascular mortality, and all-cause death (Sun et al., 2025). However, despite its growing application in cardiovascular research, the association between CTI and CCC in patients with CTO remains underexplored.

Our research demonstrates that CTI, as a marker reflecting long-term inflammatory status and metabolic stress, exhibits a significant association with an elevated risk of poor CCC, and this relationship persists robustly following multivariable adjustment. In the fully adjusted Model 3, each unit increase in CTI was associated with a 102% higher risk of poor collateral circulation (OR = 2.02, 95% CI [1.57–2.62], *P* < 0.001). The RCS analysis revealed a steady, linear increase in the risk of poor CCC with rising CTI levels, without evidence of a threshold or plateau effect. However, compared with the TyG, AIP, and NHR indices, the CTI index did not demonstrate superior discriminatory ability; its AUC was only marginally higher (0.679), and formal statistical comparison using the DeLong test confirmed that its discriminatory performance was comparable to that of the TyG index alone (*P* = 0.752). ROC analysis showed that at the optimal cut-off of 5.19, sensitivity remained relatively modest (53.9%). Although the specificity at this cut-off (74.9%) helps identify high-risk individuals, any attempt to artificially increase sensitivity by lowering the diagnostic threshold would cause a precipitous decline in specificity. In clinical practice, this steep trade-off is impractical; high false-positive rates could lead to unnecessary downstream coronary investigations, increased healthcare costs, and heightened patient anxiety. Therefore, the CTI index should be used as a complementary risk stratification tool in conjunction with traditional biomarkers and coronary imaging, rather than as a standalone screening test. Future studies could explore whether combining the CTI index with additional inflammatory or metabolic markers improves sensitivity without compromising specificity. Furthermore, formal tests for interaction revealed near-significant effect modification by smoking status (P for interaction = 0.0784). Although these interactions did not reach conventional statistical significance, they highlight clinically important patterns that warrant further investigation in larger cohorts.

In summary, we propose the CTI index as a complementary risk stratification biomarker. While its modest discriminatory power (AUC = 0.679) and relatively low sensitivity (53.9%) limit its use as a primary screen, the index retains significant prognostic value. Notably, elevated CTI remains independently associated with an increased risk of poor CCC (OR = 2.02, *P* < 0.001). As a cost-effective adjunct derived from routine admission laboratories, it should be integrated with established clinical parameters and coronary angiography to refine early risk stratification and guide individualized surveillance strategies.

## Limitations

This study has several limitations. First, it is a single-center retrospective study with a relatively small sample size, which may limit the generalizability of the findings. Second, the CTI index was measured at a single time point and may not reflect the cumulative burden or dynamic changes in metabolic inflammation. Third, detailed information on medication use (such as statins, anti-inflammatory agents, or antidiabetic drugs) was not available; these therapies can directly influence hs-CRP, triglyceride, and fasting plasma glucose levels, thereby potentially affecting CTI values and introducing residual confounding. Fourth, the findings are restricted to a Chinese population, and extrapolation to other ethnic groups should be made with caution. Finally, as this was an observational study, selection bias and unmeasured confounding cannot be entirely excluded. In subsequent research, more comprehensive longitudinal mechanisms should be further verified *via* basic experimental studies or more rigorous statistical approaches.

## Conclusions

These findings indicate a significant association between the CTI index and the risk of poor CCC in patients with CTO, while enhancing the clinical capacity for detecting poor CCC. Although these results offer initial implications that may guide subsequent preventive interventions, additional research across diverse cohorts is required to confirm and expand upon our observations.

##  Supplemental Information

10.7717/peerj.21576/supp-1 Supplemental Information 1 Full multivariable logistic regression analysis for the association between CTI index and poor coronary collateral circulation (Model 3) OR, odds ratio; CI, confidence interval; CTI, C-reactive protein–triglyceride glucose index;

10.7717/peerj.21576/supp-2 Supplemental Information 2 Pairwise comparison of ROC curves for various biomarker indices using the DeLong test Differences in the area under the curve (AUC) between CTI and other established indices for predicting poor coronary collateral circulation. Statistical significance between ROC curves was evaluated using the DeLong method. Statistically significant p-values (¡ 0.05) are highlighted in bold. AUC, area under the curve; CI, confidence interval; CTI, C-reactive protein–triglyceride glucose index; TyG, triglyceride-glucose; AIP, atherogenic index of plasma; NHR, neutrophil to HDL ratio.

10.7717/peerj.21576/supp-3 Supplemental Information 3 Raw data Raw data and data that has been separated based on Coronary Collateral Circulation (CCC) status

10.7717/peerj.21576/supp-4 Supplemental Information 4 R code

10.7717/peerj.21576/supp-5 Supplemental Information 5 STROBE checklist

## Additional Information and Declarations

### Data Availability

The following information was supplied regarding data availability: The data and code are available in the Supplemental Files.
