# Supplementary material for: Association between the C-reactive protein–triglyceride glucose index and coronary collateral circulation in patients with chronic total occlusion: a retrospective study
Source: PeerJ. 2026 Jul 20;14:e21576. doi: 10.7717/peerj.21576 (PMC13394206; doi:10.7717/peerj.21576)
Supplement: Supplemental Information 1 — OR, odds ratio; CI, confidence interval; CTI, C-reactive protein–triglyceride glucose index; [file peerj-14-21576-s001.docx]

| **Clinical and Laboratory Variables** | **OR (95% CI)** | **p value** |
| --- | --- | --- |
| CTI index | 2.016 (1.551–2.642) | <0.001 |
| Sex (male vs female) | 0.348 (0.202–0.597) | <0.001 |
| Age, years | 0.987 (0.966–1.007) | 0.202 |
| BMI, kg/m² | 1.006 (0.949–1.064) | 0.843 |
| Current smoking (yes vs no) | 1.578 (1.000–2.495) | 0.050 |
| Diabetes mellitus (yes vs no) | 1.329 (0.863–2.052) | 0.197 |
| Hypertension (yes vs no) | 1.226 (0.788–1.925) | 0.369 |
| Blood urea nitrogen, mmol/L | 0.989 (0.886–1.042) | 0.787 |
| Neutrophils, ×10⁹/L | 1.157 (1.030–1.301) | 0.014 |
| Total cholesterol, mmol/L | 1.052 (0.992–1.235) | 0.540 |
| Diastolic blood pressure, mmHg | 1.003 (0.981–1.026) | 0.771 |
| Systolic blood pressure, mmHg | 0.984 (0.970–0.998) | 0.033 |
