# Supplementary material for: Association between the C-reactive protein–triglyceride glucose index and coronary collateral circulation in patients with chronic total occlusion: a retrospective study
Source: PeerJ. 2026 Jul 20;14:e21576. doi: 10.7717/peerj.21576 (PMC13394206; doi:10.7717/peerj.21576)
Supplement: Supplemental Information 2 — Differences in the area under the curve (AUC) between CTI and other established indices for predicting poor coronary collateral circulation. Statistical significance between ROC curves was evaluated using the DeLong method. Statistically significant p-values (¡ 0.05) are highlighted in bold. AUC, area under the curve; CI, confidence interval; CTI, C-reactive protein–triglyceride glucose index; TyG, triglyceride-glucose; AIP, atherogenic index of plasma; NHR, neutrophil to HDL ratio. [file peerj-14-21576-s002.docx]

|  | **Difference of AUC** | **Standard error** | **95% CI** | ***z*-value** | ***p*-value** |
| --- | --- | --- | --- | --- | --- |
| CTI vs. TyG | 0.008 | 0.027 | -0.044–0.060 | 0.316 | 0.7521 |
| CTI vs. AIP | 0.029 | 0.028 | -0.026–0.084 | 1.041 | 0.2978 |
| CTI vs. NHR | 0.085 | 0.034 | 0.019–0.151 | 2.524 | **0.0116** |
